# The STRATOB study: design of a randomized controlled clinical trial of Cognitive Behavioral Therapy and Brief Strategic Therapy with telecare in patients with obesity and binge-eating disorder referred to residential nutritional rehabilitation

**DOI:** 10.1186/1745-6215-12-114

**Published:** 2011-05-09

**Authors:** Gianluca Castelnuovo, Gian Mauro Manzoni, Valentina Villa, Gian Luca Cesa, Giada Pietrabissa, Enrico Molinari

**Affiliations:** 1 Istituto Auxologico Italiano IRCCS, Psychology Research Laboratory, Ospedale San Giuseppe, Verbania, Italy; 2 Department of Psychology, Catholic University of Milan, Italy; 3 Department of Psychology, University of Bergamo, Italy

## Abstract

**Background:**

Overweight and obesity are linked with Binge Eating Disorder (BED). Effective interventions to significantly reduce weight, maintain weight loss and manage associated pathologies like BED are tipically combined treatment options (dietetic, nutritional, physical, behavioral, cognitive-behavioral, pharmacological, surgical). Significant difficulties with regard to availability, costs, treatment adherence and long-term efficacy are present. Particularly Cognitive Behavioral Therapy (CBT) is the therapeutic approach indicated both in in-patient and in out-patient settings for BED. In recent years systemic and systemic-strategic psychotherapies have been implemented to treat patients with obesity and BED involved in familiar problems. Particularly a brief protocol for the systemic-strategic treatment of BED, using overall the strategic dialogue, has been recently developed. Moreover telemedicine, a new promising low cost method, has been used for obesity with BED in out-patient settings in order to avoid relapse after the in-patient step of treatment and to keep on a continuity of care with the involvement of the same clinical in-patient team.

**Methods:**

The comparison between CBT and Brief Strategic Therapy (BST) will be assessed in a two-arm randomized controlled clinical trial. Due to the novelty of the application of BST in BED treatment (no other RCTs including BST have been carried out), a pilot study will be carried out before conducting a large scale randomized controlled clinical trial (RCT). Both CBT and BST group will follow an in-hospital treatment (diet, physical activity, dietitian counseling, 8 psychological sessions) plus 8 out-patient telephone-based sessions of psychological support and monitoring with the same in-patient psychotherapists. Primary outcome measure of the randomized trial will be the change in the Global Index of the Outcome Questionnaire (OQ-45.2). Secondary outcome measures will be the percentage of BED patients remitted considering the number of weekly binge episodes and the weight loss. Data will be collected at baseline, at discharge from the hospital (c.a. 1 month after) and after 6-12-24 months from the end of the in-hospital treatment. Data at follow-up time points will be collected through tele-sessions.

**Discussion:**

The STRATOB (Systemic and STRATegic psychotherapy for OBesity), a comprehensive two-phase stepped down program enhanced by telepsychology for the medium-term treatment of obese people with BED seeking intervention for weight loss, will shed light about the comparison of the effectiveness of the BST with the gold standard CBT and about the continuity of care at home using a low-level of telecare (mobile phones).

**Trial registration:**

ClinicalTrials.gov Identifier: NCT01096251

## Background

One of the most important medical and public health problems of our time is represented by obesity [1]. It is widely considered as a chronic pathology and a risk factor for many medical complications such as endocrinological, pneumological, cardiovascular, and orthopedic diseases [2-8]. Overweight and obesity are also traditionally connected with Binge Eating Disorder (BED), psychopathology characterized by frequent and persistent episodes of binge eating with the key feeling of losing the control and the significative distress due to the absence of regular compensatory behaviors such as in the patients with bulimia [9]. Effective interventions to significantly reduce weight, maintain weight loss and reduce related pathologies like BED are tipically combined treatment options (dietetic, nutritional, physical, behavioral, cognitive-behavioral, pharmacological, surgical). Significant difficulties in these kinds of approaches have been underlined with regard to availability, costs, treatment adherence and long-term efficacy [10]. Even if the combined treatment options are the gold standard in weight loss, most overweight and obese individuals regain about one third of the weight lost with treatment within 1 year [11] and they will typically come back to baseline in 3 to 5 years [12-14].

About the psychological treatment of BED, Cognitive Behavioral Therapy (CBT) and Interpersonal Psychotherapy (IPT) are the psychotherapies more indicated in the Eating Disorder field [9]. Particularly CBT is the therapeutic approach indicated both in in-patient and in out-patient settings for BED [15-27].

The prevalence and spread of CBT is not under discussion, in recent years systemic, strategic and systemic-strategic psychotherapies have been implemented to treat patients with obesity and BED, overall when the pathology includes familiar characteristics and problems [26-30]. Particularly a brief protocol for the systemic-strategic treatment of BED, using overall the typical brief techniques such as the strategic dialogue, has been recently developed by Nardone and Portelli in the Handbook of Brief Strategic Therapy (BST) [31] and other contributions [32,33].

In order to deal with problems of weight regain and treatment relapses after 12-24-36 months, telemedicine, a new promising low cost method, has been used for obesity with BED in out-patient settings. This enhances the treatment after in-patients rehabilitation and keeps continuity of care through the involvement of the same clinical in-patient team [34-37].

Taking into account this scenario, we developed the STRATOB study (*Systemic and STRATegic psychotherapy for OBesity*), a comprehensive two-phase stepped down program enhanced by telepsychology for the medium-term treatment of obese people with BED seeking intervention for weight loss. The key aspects of the STRATOB study are the hospital-based intensive treatment and the continuity of care at home using a low-cost level of telecare (mobile phones). Many treatments delivered using new technologies, such as web-sites, e-mails, chat lines, videoconferences, UMTS mobile-phones and traditional telephones could represent a valid integration for traditional psychotherapy reducing expensive and time-consuming clinical visits and improving adherence to prescribed psychological, dietetic and medical treatments through extended monitoring and support [10,12,34,38-46].

This paper describes the design of the STRATOB study, a small scale two-arm randomized controlled clinical trial (RCT). The main aim of this study will be to compare the effectiveness of the BST [31-33] with the gold standard CBT [26,47-53] in a in-patient and telephone-based out-patient program for a sample of obese people with BED seeking treatment for weight reduction.

## Methods

The comparison between the CBT and BST will be assessed in a two-arm randomized controlled clinical trial. Participants will be randomly allocated in 2 groups:

1) CBT group: in-hospital treatment (diet, physical activity, dietitian counseling, 8 sessions of CBT) plus 8 out-patient telephone-based sessions of CBT-oriented psychological support and monitoring with the same CBT in-patient psychotherapists;

2) BST group: in-hospital treatment (diet, physical activity, dietitian counseling, 8 sessions of BST) plus 8 out-patient telephone-based sessions of BST-oriented psychological support and monitoring with the same BST in-patient psychotherapists;

The Medical Ethics Committee of Istituto Auxologico Italiano approved the study protocol and the Informed Consents.

The rehabilitation program considered in this study will have a total duration of 7 months and consists of two stepped down phases: in-patient (1 month) and out-patient (the following 6 months).

During the in-patient phase, participants will undergo an intensive four-week hospital-based and medically-managed rehabilitation program for weight reduction. Along this period, participants will live in a hospital located on a mountain highland and far away from towns and cities. Visits from family members and friends are allowed only in the afternoon. All patients will be placed on a hypocaloric nutritionally balanced diet tailored to the individual after consultation with a dietitian (energy intake around 80% of the basal energy expenditure estimated according to the Harris-Benedict equation and a macronutrient composition of about 16% proteins, 25% fat and 59% carbohydrates). Furthermore, they will receive nutritional counseling provided by dietitians, psychotherapy provided by psychologists-psychotherapists (well trained in CBT and BST approaches) and have physical activity training provided by physiotherapists.

Nutritional rehabilitation program aims to improve and promote change in eating habits and consists of both individual sessions (dietary assessment, evaluation of nutrient intake and adequacy, nutritional and anthropometric status, eating patterns, history of overweight, readiness to adopt change) and group sessions (45 minutes each session, twice a week, including: information on obesity and related health risks, setting of realistic goals for weight loss, healthy eating in general, general nutrition and core food groups, weight management and behavior change strategies for preventing relapse).

Psychotherapy will be provided twice a week in individual setting. CBT individual sessions, lasting 45 minutes each, are mainly based on the cognitive-behavioral approach described by Cooper and Fairburn [53] and emphasize the techniques of self-monitoring, goal setting, time management, prompting and cueing, problem solving, cognitive restructuring, stress management and relapse prevention. BST individual sessions, lasting 45 minutes each, are mainly based on the brief strategic approach described by Nardone and colleagues [31-33] and emphasize the techniques of working on "attempted solutions" (such as keeping control by abstaining from food and continuously striving to exert self-control with a subsequent loss of control), using reframing maneuvers, inducing fear of fasting rather then bingeing, understanding what is maintaining and worsening the problem, etc. (p. 105-105, [31]).

Physical activity will take place once a day except for week-end and consists of group programs (20 subjects) based on postural gymnastics, aerobic activity and walks in the open. In-patients with specific orthopedic complications will carry out individual activities planned by physiotherapists and articulated in programs of physical therapy, assisted passive and active mobilization and isokinetic exercise.

At the end of the in-patient step low to moderate weight losses will be expected and it is important to underline that weight loss is not the primary goal of the in-patient program (each patient is made clear about this point at the very beginning of the treatment). Beyond the medical management of metabolic risk factors for health such as type 2 diabetes, developing a sense of autonomy and competence will be the primary purposes of the in-hospital interventions. Skills and tools for change will be provided to patients and they will be supported in assigning positive values to healthy behaviors and also in aligning them with personal values and lifestyle patterns.

Just after discharge, participants will have 4 telephone calls with their in-patient psychotherapists along 2 months. From the 3rd to the 6th month sessions will be scheduled every 30 days. During tele-sessions, psychotherapists will test the out-patients' progress, their mood, the maintenance of functional alimentary and physical activity habits, the loss/increase of weight and will discuss about critical moments. Some research indicates that changes in behavior (eating and exercise) often follow discrete moments which have been variably described as life events or life crises [54]. Life events can lead to weight loss but also to weight gain and qualitative research shows that it is not the event per se that results in behavior change but the ways in which this event is appraised, interpreted and managed by the individual [55]. Psychotherapists (both CBT and BST) will have thus the opportunity to discuss with their patients in remote settings about the significant events during the telephone calls and will cognitively reconstruct dysfunctional appraisals in functional ways. In particular, phone-sessions with the psychotherapists will aim to consolidate strategies and abilities acquired during the in-patient phase, to improve self-esteem and self-efficacy, to support motivation, to prevent relapse and to provide problem-solving and crisis counseling.

As underlined, in the out-patient step of the STRATOB program great relevance will be given to the clinician-patient relationship as an important medium and vehicle of change [56]. After discharge, out-patients will begin to experience the autonomy and competence to change they will have developed during the in-patient phase and inevitably face resistances and barriers. Out-patients will be supported by telephone by the psychotherapists who will have attended them during the in-hospital phase in exploring resistances and barriers they experience and in finding functional pathways to cope. Furthermore, out-patients will be helped to experience mastery in terms of the health behavior change that needs to be engaged.

### Recruitment of the study population

One hundred consecutive female in-patients with obesity and BED in the age range of 18-65 years, who will be referred to a single clinical center (Saint Joseph Hospital - Istituto Auxologico Italiano IRCCS) for weight-loss treatment, will be asked and screened for admission to the study. Patients will be sequentially recruited.

### Inclusion and exclusion criteria

In-patients will be eligible when they will meet the following inclusion criteria at the admission to the hospital: 1) age between 18 and 65 years; 2) obesity according to the WHO criteria (BMI ≥ 30); 3) BED (DSM-IV-TR criteria) and 4) written and informed consent to participate. Exclusion criteria for the study are: 1) other severe psychiatric disturbance diagnosed by DSM-IV-TR criteria; 2) concurrent medical condition not related to obesity. SCID (Structured Clinical Interview for DSM-IV-TR Disorders) I and II [57] will be used as screening tools for psychiatric disorders and will be administered by an independent clinical psychologist as part of his work.

### Randomization procedure

All participants will be randomly assigned to the CBT or BST group. The randomization scheme will be generated using the Web site Randomization.com (http://www.randomization.com). Randomization will occur after the baseline measurements.

### Patient Assessment and Measurements

Primary outcome measure of the randomized trial will be the change in the Global Index of the Outcome Questionnaire (OQ 45.2).

Secondary outcome measures will be the percentage of BED patients remitted considering the number of weekly binge episodes and the weight loss. Data will be collected at baseline, at discharge from the hospital (c.a. 1 month after) and after 6-12-24 months from the end of in-hospital treatment.

Weight will be assessed with the participant in lightweight clothing with shoes removed on a balance beam scale.

Data collected at follow-up time points will be collected through tele-sessions.

### Psychological and behavioral assessment

Participants will complete the Outcome Questionnaire (OQ 45.2) - Italian translation and validation [58,59] at entry to the study and at discharge from the hospital with a self-report procedure. The number of weekly binge episodes will be assessed with a self-report procedure at entry to the study and at discharge from the hospital. At 6-12-24 month follow-up data will be collected via telephone during the tele-sessions.

### Outcome Questionnaire (OQ 45.2)

The OQ 45.2 is a self report questionnaire developed by Michael Lambert in 1996 [60]. The OQ 45 items version is a measure of outcome and it is designed in order to collect repeated measures of patient progress during psychotherapy and after its conclusion. This instrument is one of the most used in psychotherapy research in the U.S. [61]. The OQ 45.2 is composed by 45 items that form 3 scales: Symptom Distress (SD), Interpersonal Relations (IR) e Social Role (SR), and a Global Index. The OQ 45.2 is proposed as a brief screening and outcome scale that tries to assess the subjective experience of a person, as well as the way he or she functions in the world [60]. The choice of this questionnaire has to take into account the need to measure a change in a short time (1 month of in-patient treatment) due to psychotherapeutic activities. The Italian version of the OQ-45.2 appears promising as a measure of general psychological distress, and it could be used to measure the psychotherapy outcome in routine clinical practice. So the Outcome Questionnaire-45 was developed as a brief measure of client progress in psychotherapy to address the need for accountability in the era of managed care and to improve upon extant outcome measures.

The investigator involved in administering and interpreting the OQ45.2 will be blinded to treatment assignment.

### Psychotherapists and Treatment Fidelity

Treatments will be delivered by 4 experienced and certified psychotherapists from diverse backgrounds with specific training in CBT and BST. Psychotherapists will receive monthly supervision by senior certified psychotherapists to ensure competent and uniform treatment delivery.

### Sample size calculation and statistical analysis

Due to the novelty of the application of BST in BED treatment, no other RCTs including BST have been carried out. So this RCT will be similar to a pilot study, typically defined as small scale preliminary study, and according to Lackey and Wingate [62], a pilot work may use at least the 10% of the sample required. Using an A-priori Sample Size Calculator for Student's t-Test (G*Power 3.1.2 software), 394 participants per group will be needed to detect a difference with an estimated Effect Size (Cohen's d) of 0.2, an alpha of 0.05 two-sided, a desired statistical power of 0.80. We decided not to make assumptions on sidedness not having previous results about comparison between CBT and BST. Taking into account the need of at least 10% of the sample required (788 individuals, n = 394 per group), 80 persons (n = 40 per group) will be considered enough.

Weight data will be analyzed with an intention-to-treat (ITT) approach with dropouts assumed to have regained 0,3 kg per month, an assumption already used in previous studies [12,63]. Differently, missing data in the other variables will be replaced with baseline observation carried forward (BOCF) or last observation carried forward (LOCF) as appropriate, assuming no improvement for non-responders patients.

The independent samples t test will be used to examine between-group differences in OQ variables and weight at all time-points. Change scores will be also examined. Chi-square will be used to test the association between treatment groups and number of weekly binge episodes ≥ 2 at 6 months. Corrected effect sizes (Hedges) and 95% Confidence Interval will be calculated for both between-group and with-in group differences.

All data analyses will be performed using the Statistical Package for the Social Sciences (version 16.0; SPSS, Inc., Chicago, IL).

## Discussion

Due to the novelty of Brief Strategic Therapy (BST) in the treatment of Binge Eating Disorders, no well defined hypothesis have been established about the possible contribution of this kind of therapy in comparison with Cognitive Behavior Therapy (CBT), the "gold standard" psychological treatment for BED. With the development of a brief systemic-strategic clinical protocol for treatment of BED [31-33], new interventions in the field of eating disorders are available and have to be evaluated. Systemic and systemic-strategic psychotherapies such as BST traditionally focus on systemic-family problems and contexts and these areas of interventions could represent a real added value in comparison with CBT. Moreover, BST sessions could emphasize the techniques of working on "attempted solutions", using reframing maneuvers, inducing fear of fasting rather then bingeing, understanding what is maintaining and worsening the problem, etc. (p. 105-106, [31]). This focus on "attempted solutions", that in the typical BED are related to dysfunctional habits such as "trying to keep control by abstaining from food and continuously striving to exert self-control with a subsequent loss of control", could generate some differences between the BST and the CBT approach about the remission of weekly binge episodes at 6-12-24 months (during the in-patient phase patients will do not cope with the binge temptations due to the lack of them in a safe place like the hospital). Moreover the focus of BST on symptoms reduction (in particular anxiety and depression) in short time (traditionally 6-12 months) could generate significant difference between CBT and BST in the OQ 45.2 Symptom Distress (SD) scale.

The real therapeutic impact of CBT and BST could be significantly reduced by the use of a non-traditional setting in the second part of the trial. Even if telecare could represent a great opportunity for the continuity of treatments "moving the healthcare where it really needs", the limitations of non-verbal communications, due to the use of telephone sessions, could influence the CBT and BST protocols in different ways that have to be investigated.

A possible limitation in this trial is the small sample size. Even if this is the first study that compared CBT with BST in the treatment of BED, larger clinical trials will be needed in order to evaluate both the effectiveness of integrated psychotherapies (such as the traditional CBT combined with BST or IPT) and the use of new technologies in the clinical field (from the "low technology" telephone to the "medium technology" clinically oriented SMS, MMS, e-mail, chat, web sources, till the "high technology" web 2.0 or virtual and augmented realities) [64-70].

Moreover generalization of research findings will be limited because the STRATOB trial will be a single-centre randomized controlled study. Future multi-centre trials should extend both the use of alternative models of psychological support or psychotherapy, i.e. BST, and the implementation of new technologies, in order to find the best combination of the variables involved (psychotherapeutic model, level of technology, number of sessions, etc.).

Taking into account that traditional interventions for BED patients with obesity typically fails because of low compliance with clinical programs, continuous monitoring and psychological support are needed. This study will improve the evidence-based knowledge of how CBT and BST may enhance the long-term efficacy of clinical interventions for BED and weight loss using telecare settings too.

Due to the growing development of technologies in the clinical applications, we are moving towards a new "health care everywhere" approach, where clinicians could provide their treatments in both traditional clinical settings (public and private hospitals, clinics, services, etc.) and innovative clinical settings (remote out-patients' clinics, tele-health and e-health based settings) [65,68,70-72].

## List of abbreviations used

BED: Binge Eating Disorder; CBT: Cognitive Behavioral Therapy; IPT: Interpersonal Psychotherapy; BST: Brief Strategic Therapy; OQ: Outcome Questionnaire; RCT: Randomized Clinical Trial; STRATOB: Systemic and STRATegic psychotherapy for OBesity; SMS: Short Message Service; MMS: Multimedia Messaging Service; SD: Symptom Distress; IR: Interpersonal Relations; SR: Social Role; UMTS: Universal Mobile Telecommunications System; SCID: Structured Clinical Interview for DSM Disorders; DSM: Diagnostic and Statistical Manual of Mental Disorders.

## Competing interests

The authors declare that they have no competing interests.

## Authors' contributions

GC conceived the study, planned its design and made substantial contribution to the manuscript drafting. GMM participated in the study design and contributed to the manuscript drafting. VV, GLC, GP and EM participated in the study design and helped to draft the manuscript. All authors read and approved the final manuscript.
